# Trichoblastic Carcinoma of the Right Clavicular Region: A Case Report With a Review of the Literature

**DOI:** 10.7759/cureus.82797

**Published:** 2025-04-22

**Authors:** Kavita Jadhav, Rajalakshmi Venkateswaran, Rukmini Waghmare, Girish Bakhshi, Yogesh Jaiswal

**Affiliations:** 1 General Surgery, Grant Government Medical College and Sir J.J. Group of Hospitals, Mumbai, IND; 2 Plastic and Reconstructive Surgery, Grant Government Medical College and Sir J.J. Group of Hospitals, Mumbai, IND

**Keywords:** excision of part of clavicle, skin adnexal malignancy, trapezius flap, trichoblastic carcinoma, wide local excision

## Abstract

Trichoblastic carcinoma is a rare skin adnexal malignancy. It often resembles other skin malignancies in its gross and histopathological picture. While the definitive treatment for low-grade lesions is wide local excision, high-grade or inoperable lesions require chemo-radiation. It is important to ensure excision of an adequate clear skin margin while doing a surgical resection.

The present case reports a 75-year-old male with a malignant skin lesion on his right clavicle. Magnetic resonance imaging showed it to involve the muscles and clavicle. Preoperative biopsy showed features of a trichilemmal carcinoma.

A wide local excision of the part of the clavicle was done. A trapezius flap was mobilized to cover the defect. Histopathology and immunohistochemistry analysis confirmed the diagnosis of a low-grade trichoblastic carcinoma.

This case report aims to emphasize that trichoblastic carcinoma can be considered one of the differentials of skin adnexal malignancies, and it needs a multidisciplinary approach to manage such rare malignancies.

## Introduction

Skin adnexal malignancies arise from adnexal structures of the skin such as hair follicles and sweat or sebaceous glands. Trichoblastic carcinoma (TBC) is one such tumor that can arise either de novo or due to the morphological differentiation of a trichoblastoma [1]. It is a rare entity, with only less than 100 cases reported till 2022 in the literature [2].

The most common site for occurrence is the head and neck, accounting for nearly 50-60% of all cases [2]. High-grade tumors and patients with nodal metastasis have a high risk of recurrence. Its gross and microscopic appearance often mimics other cutaneous cancers, making it difficult to diagnose pre-operatively, as in the present case.

The presence of hypercellular stroma is a distinguishing feature from basal cell carcinoma, and immunohistochemistry helps differentiate TBC from other skin adnexal malignancies [3,4]. 

Surgical removal of large lesions involving the muscle, bone etc. often requires to be followed by composite flaps and bone reconstruction to bridge the defect and ensure good postoperative function and cosmesis. Chemo-radiation may be required postoperatively.

## Case presentation

A 75-year-old man presented with a non-healing ulcer over the right mid-clavicular region for one year following the excision of a soft tissue swelling from the same site one year ago. The patient was a known case of bronchial asthma. He had no history of any addiction. He was a fruit vendor and used to sit in the sunlight to sell them.

**Figure 1:**
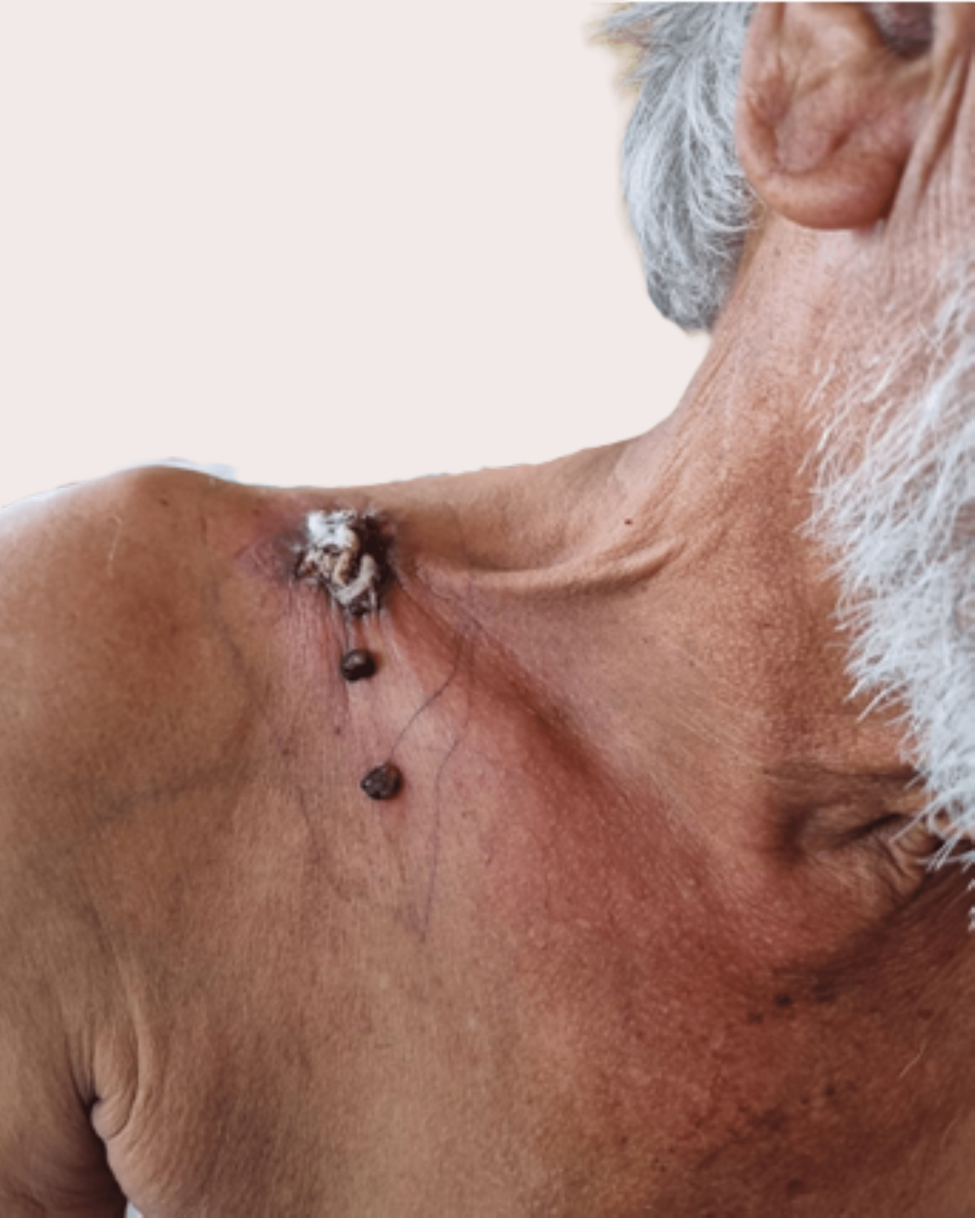
Primary lesion with two satellite nodules

Local examination revealed a 2x1 cm ulceroproliferative growth over the medial and lateral one-third of the clavicle with black pigmented nodules over it. The growth was non-mobile and bled on touch. Surrounding redness and induration were present for 1 cm, and platysma contracture was noted. Additionally, two black nodules were noted below and anterior to the above lesion, each measuring approximately 1 x 1 cm as shown in the image (Figure 1).

**Figure 2:**
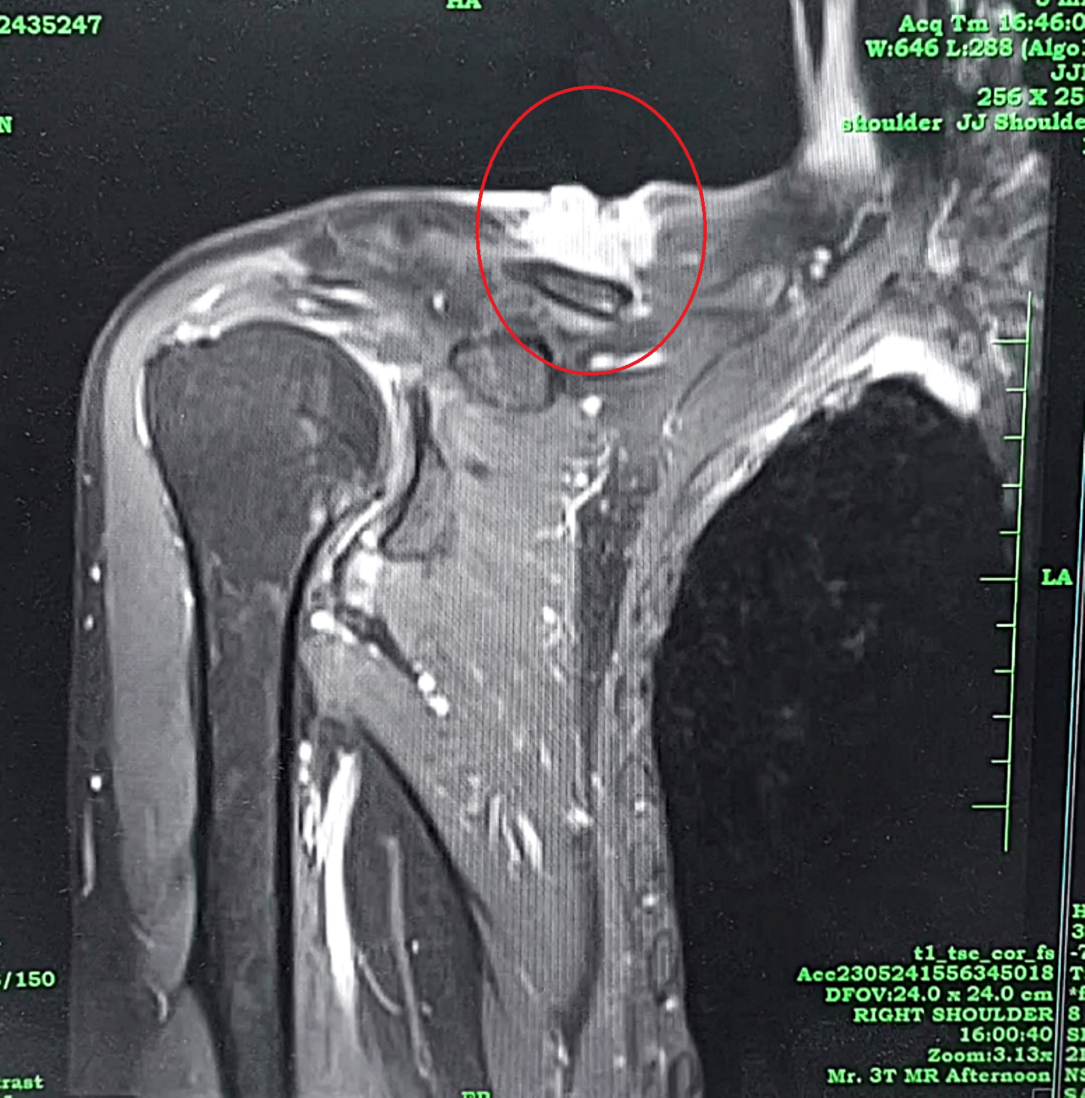
MRI STIR image showing a hyper-intense signal at the site of lesion STIR: short tau inversion recovery

No other lesion was noted on head-to-toe examination. MRI neck was done, which revealed a neoplastic mass with extension into the adjacent trapezius and deltoid muscle fibers and short tau inversion recovery (STIR) hyperintense signal in the distal third of the clavicle showing post-contrast enhancement suggestive of marrow invasion (Figure 2).

**Figure 3:**
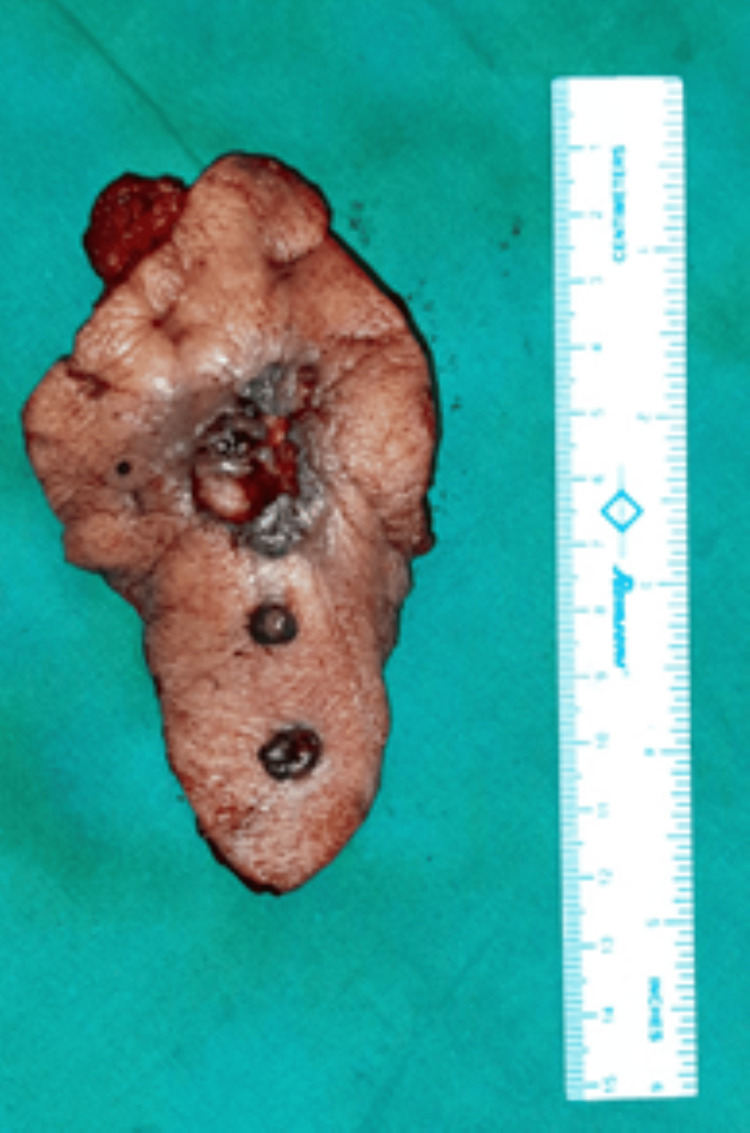
Resected specimen

A positron emission tomography (PET) scan ruled out any evidence of distant metastasis. An edge wedge biopsy was suggestive of trichilemmal carcinoma. The patient was posted for wide local excision (WLE) with a flap cover under general anesthesia. An elliptical incision involving all three lesions was made. The horizontal extent of excision was 1 cm beyond the farthest point of induration (Figure 3).

**Figure 4:**
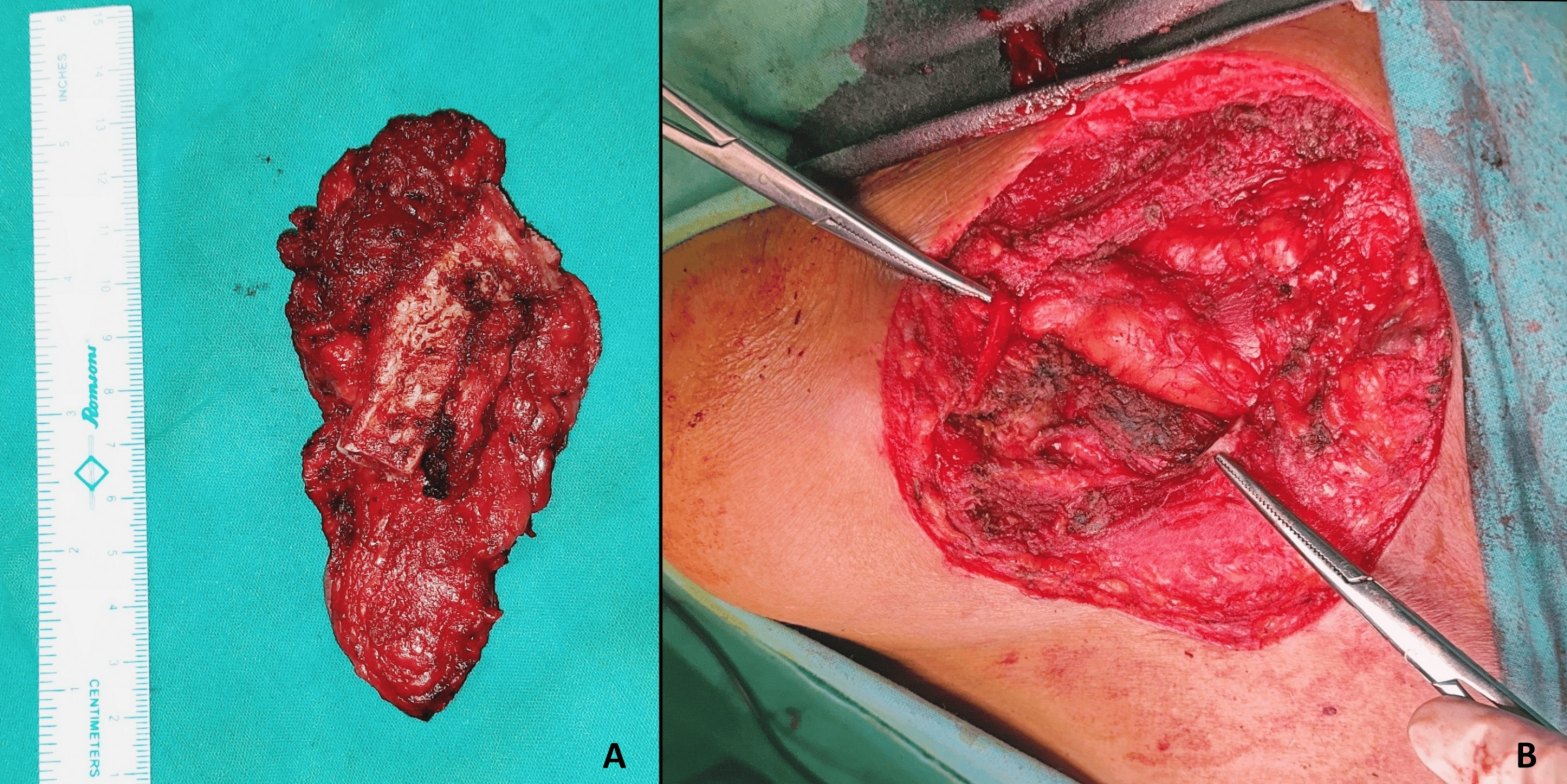
Images explaining clavicle excision A: the resected specimen; B: preserved ends of the clavicle

Parts of the deltoid and trapezius involved by the tumor were shaved with a clear gross margin of 1 cm. Around 4-5 cm of the lateral aspect of the clavicle was excised in toto, leaving behind the lateralmost end, as it was not involved clinically, which was also confirmed radiologically. It ultimately helped to retain the mobility of the shoulder without disturbing the joint and excluded the need for clavicular reconstruction (Figures 4A-4B).

**Figure 5:**
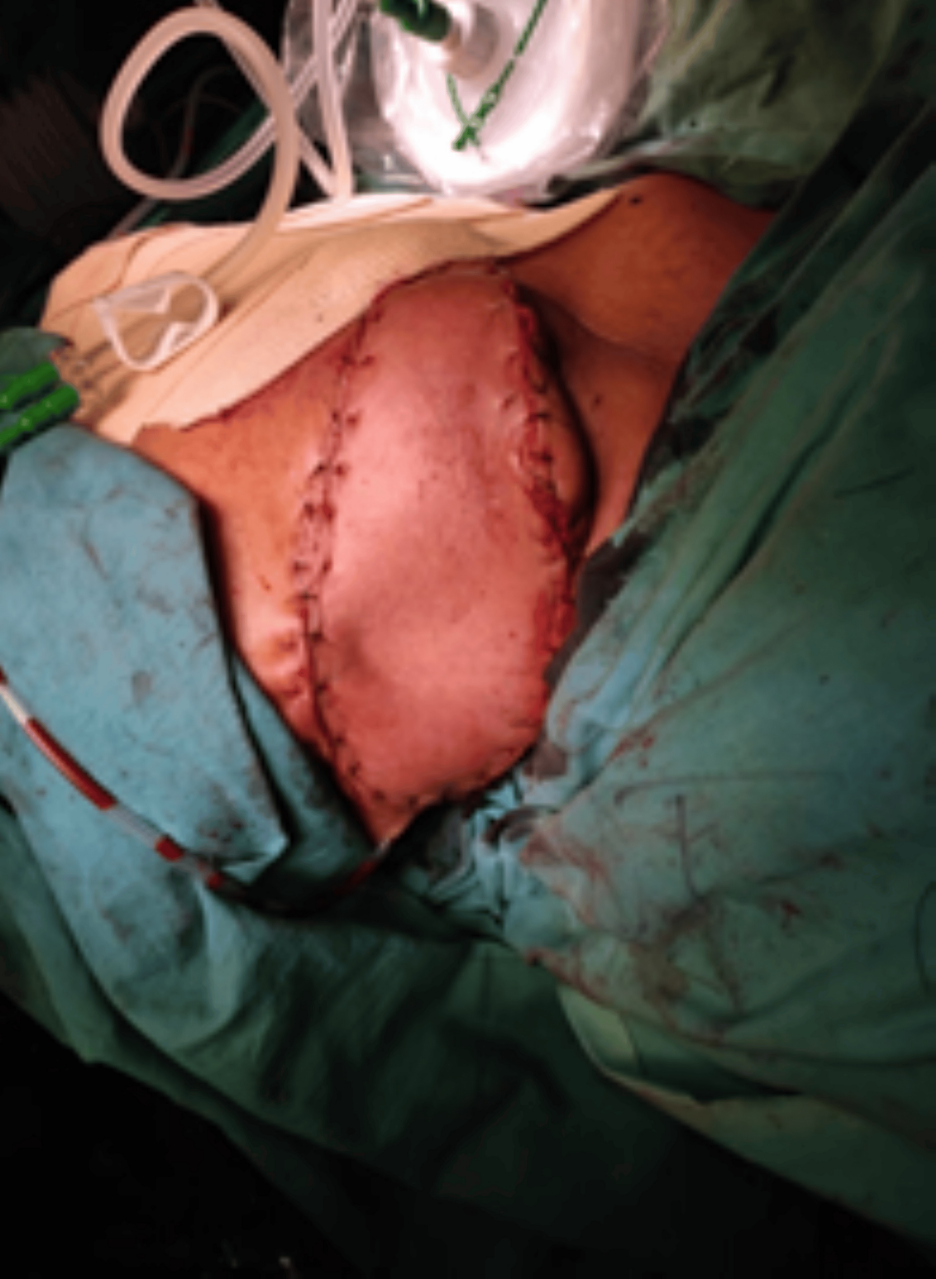
A pedicled trapezius flap covering the defect

Subsequently, with the assistance of plastic surgeons, a pedicled trapezius flap was mobilized, and the defect was covered (Figure 5). The arm was strapped firmly to the chest.

Postoperatively, the patient was nursed in the left lateral position for five days, and dressings were changed every alternate day. Meanwhile, movements of the fingers, wrist joint, and elbow were initiated. Physiotherapy for the shoulder joint was started on postoperative day 14.

**Figure 6:**
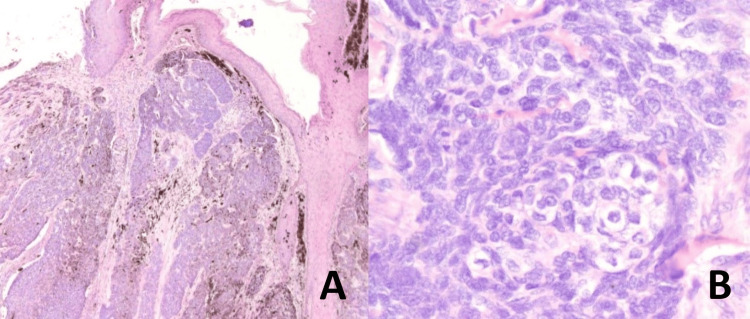
Histopathology images A & B: islands of tumor cells in hair follicles and fusiform and central clear cells, cellular stroma, and enlarged nuclei and papillary mesenchymal bodies

The histopathology report revealed a diagnosis of low-grade trichoblastic carcinoma (TBC), with papillary mesenchymal bodies (Figures 6A-6B).

**Figure 7:**
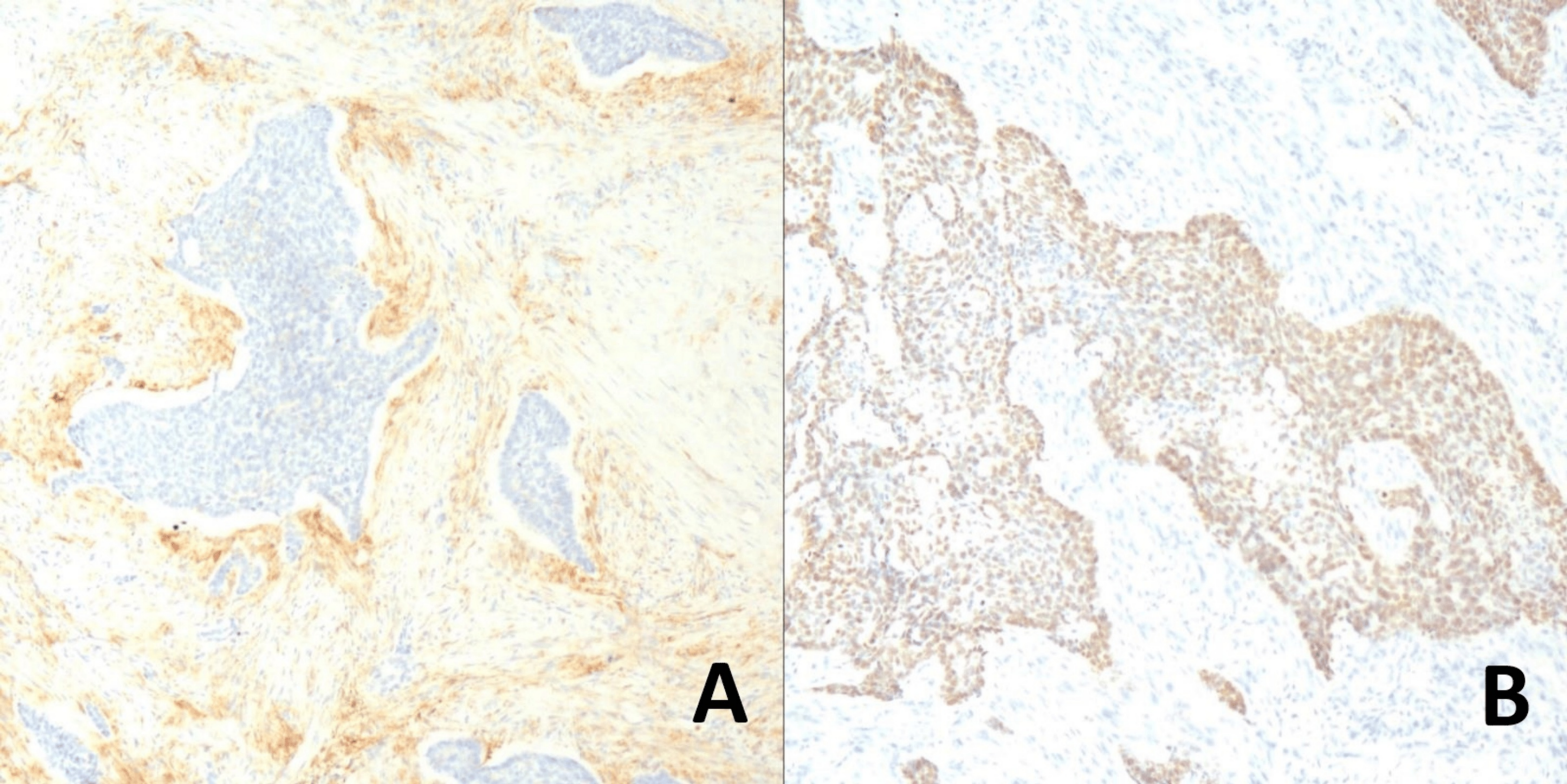
Immunohistochemistry analysis A: Cells showing focal immune-positivity for p40/p63/CK 5 & 6/HMWCK; B: Cells showing immune-positivity for cytokeratin 20/CD10

The margins were free of tumor, suggestive of R0 resection. On immunohistochemistry (IHC), the cells showed focal positivity for CK20/CD10, p40, p63, CK 5 & 6, and HMWCK (Figures 7A-7B) and were immune-negative for BerEP4.

**Figure 8:**
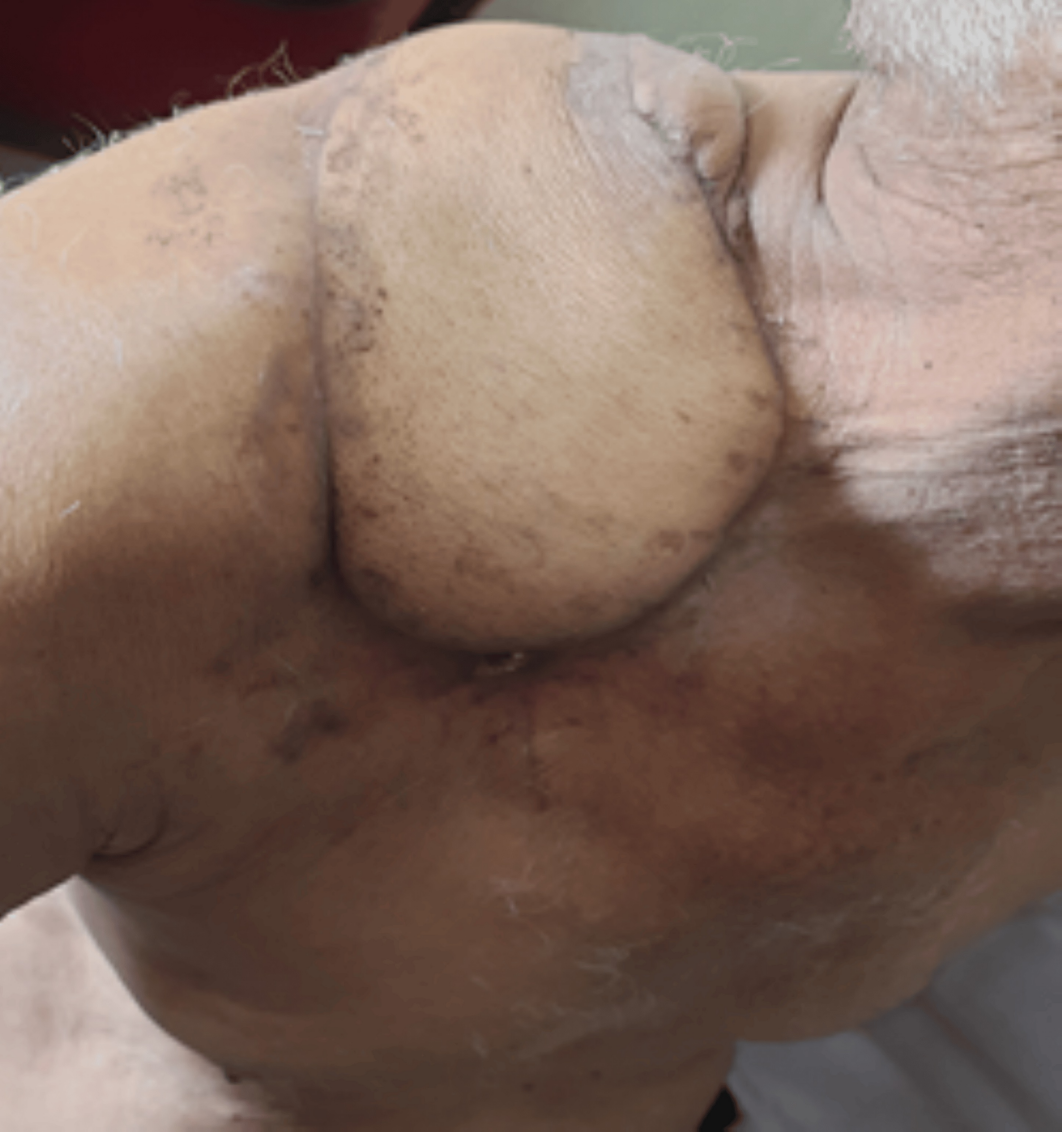
Healthy scar

The suture line healed well (Figure 8).

At the time of discharge, the range of shoulder joint movements was assessed. The patient was able to abduct the upper limb up to 160 degrees. Flexion, extension, and internal and external rotatory movements were free and full. He was able to carry out daily activities of personal care.

The patient received 25 radiation cycles locally. After this, he was able to work in his shop comfortably, without pain or restriction of movement. However, he was not able to lift heavy weights or push his vending truck. On doing a repeat PET scan after one year, there was no evidence of recurrence.

## Discussion

Trichoblastic carcinoma was first described in the literature by Headington and French in 1962 [1]. Due to its ability to mimic other cutaneous malignancies, it is often difficult to diagnose before biopsy and HPE report.

BCC is invasive but rarely metastatic and originates in hair follicle-derived cells or interfollicular zones of the epidermis. Trichoblastoma (TB) is an infrequent benign skin neoplasm that differentiates into follicular germinative cells. The presence of papillary mesenchymal bodies, cellular stroma, keratin cysts, and clefts between the epithelial-stromal tumor and dermis differentiates TBC from BCC [5].

IHC for TBC shows positivity for CK20 (Merkel cells), and negative for androgen receptors, which is opposite to BCC [4]. IHC for TBC also shows positivity for CD10, cytokeratin 20, CK 5 and 6, which are negative in other skin cancer cells [3,4].

A comprehensive review of literature presented by Michelle A. Boettler and colleagues has shown surgery to be the ideal primary modality of management [2]. Out of 93 cases, 82.8% required only surgery, as they were low to intermediate grade lesions with no metastasis. Additionally, of the 40 operated cases on follow-up, no recurrence was noted after 28 months in 87.5% [2].

In the present case, the terminal end of the clavicle forming the girdle was not resected in WLE. So, the basic functions of the joint were expected to be preserved. This can be explained by the fact that the clavicle forms a part of the shoulder girdle, but the movements of the shoulder joint occur at the glenohumeral joint, which comprises parts of the scapula and humerus.

Additionally, the acromioclavicular joint at the lateral end of the clavicle prevents excessive rotation of the scapula and strengthens the joint capsule, thus maintaining joint mobility [6].

Due to the rarity of TBC, the role of radiotherapy (RT) has not been clearly defined. However, RT has been shown to be beneficial in prolonging survival and preventing progression in inoperable cases, recurrence, positive surgical margins, and high-grade lesions of TBC [7,8].

Such cases actively require a multidisciplinary approach along with psychological counseling, nutritional and cardiopulmonary optimization, surgery, reconstruction, post-procedure physiotherapy, and chemo-radiotherapy.

## Conclusions

Trichoblastic carcinoma can mimic other skin malignancies in its gross and microscopic appearance. Hence, it is important to rule out more common conditions before making a diagnosis. IHC plays a crucial role in diagnosing such rare malignancies, and treatment approaches should be individualized.

Skin adnexal malignancies can be managed by wide local excision, ensuring adequate negative margins, with or without reconstruction, followed by active physiotherapy and postoperative chemo-radiation. Thus, a multidisciplinary approach is a must for all malignancies, including skin adnexal tumors.
